# 

**Published:** 1864-01

**Authors:** 


### BOOKS RECEIVED.

*Outlines of the Chief Camp Diseases of the United States Armies, as observed during the present War—a Practical Contribution to Military Medicine*—By Joseph Janvier Woodward, M. D., *Assistant Surgeon U. S. A.; Member of the Academy of Natural Sciences of Philadelphia; of the Pathological Society of Philadelphia, etc., etc. Philadelphia:* J. B. Lippincott & Co., 1863.

For sale by Breed, Butler & Co.

*A Manual on Extracting Teeth; founded on the Anatomy of the parts involved in the operation; the kinds and proper construction of the instruments to be used; the accidents liable to occur from the operation, and the proper remedies to retrieve such accidents.* By ABRAHAM ROBERTSON, D. D. S., M. D., *author of Prize Essay on Extracting Teeth, etc. Philadelphia:* LINDSAY & BLAKISTON, 1863.

For sale in Buffalo by BREED, BUTLER & CO.. Price $1.50.

